# Remodeling of Intracellular Ca^2+^ Homeostasis in Rat Hippocampal Neurons Aged In Vitro

**DOI:** 10.3390/ijms21041549

**Published:** 2020-02-24

**Authors:** Maria Calvo-Rodriguez, Elena Hernando-Pérez, Sara López-Vázquez, Javier Núñez, Carlos Villalobos, Lucía Núñez

**Affiliations:** 1 Alzheimer Research Unit, Department of Neurology, Massachusetts General Hospital and Harvard Medical School, Charlestown, MA 02129, USA; 2 Institute of Biology and Molecular Genetics (IBGM), University of Valladolid and National Research Council (CSIC), 47003 Valladolid, Spain; elena.hernando@ibgm.uva.es (E.H.-P.); saralpzvz@gmail.com (S.L.-V.); carlosv@ibgm.uva.es (C.V.); nunezl@ibgm.uva.es (L.N.); 3 Faculty of Odontology, Complutense University of Madrid, 28040 Madrid, Spain; javilds@hotmail.com; 4 Department of Biochemistry and Molecular Biology and Physiology, University of Valladolid, 47005 Valladolid, Spain

**Keywords:** calcium, hippocampal neurons, aging, Alzheimer’s disease, amyloid beta oligomers, endoplasmic reticulum, mitochondria

## Abstract

Aging is often associated with a cognitive decline and a susceptibility to neuronal damage. It is also the most important risk factor for neurodegenerative disorders, particularly Alzheimer’s disease (AD). AD is related to an excess of neurotoxic oligomers of amyloid β peptide (Aβo); however, the molecular mechanisms are still highly controversial. Intracellular Ca^2+^ homeostasis plays an important role in the control of neuronal activity, including neurotransmitter release, synaptic plasticity, and memory storage, as well as neuron cell death. Recent evidence indicates that long-term cultures of rat hippocampal neurons, resembling aged neurons, undergo cell death after treatment with Aβo, whereas short-term cultures, resembling young neurons, do not. These in vitro changes are associated with the remodeling of intracellular Ca^2+^ homeostasis with aging, thus providing a simplistic model for investigating Ca^2+^ remodeling in aging. In vitro aged neurons show increased resting cytosolic Ca^2+^ concentration, enhanced Ca^2+^ store content, and Ca^2+^ release from the endoplasmic reticulum (ER). Ca^2+^ transfer from the endoplasmic reticulum (ER) to mitochondria is also enhanced. Aged neurons also show decreased store-operated Ca^2+^ entry (SOCE), a Ca^2+^ entry pathway related to memory storage. At the molecular level, in vitro remodeling is associated with changes in the expression of Ca^2+^ channels resembling in vivo aging, including changes in N-methyl-D-aspartate NMDA receptor and inositol 1,4,5-trisphosphate (IP_3_) receptor isoforms, increased expression of the mitochondrial calcium uniporter (MCU), and decreased expression of Orai1/Stim1, the molecular players involved in SOCE. Additionally, Aβo treatment exacerbates most of the changes observed in aged neurons and enhances susceptibility to cell death. Conversely, the solely effect of Aβo in young neurons is to increase ER–mitochondria colocalization and enhance Ca^2+^ transfer from ER to mitochondria without inducing neuronal damage. We propose that cultured rat hippocampal neurons may be a useful model to investigate Ca^2+^ remodeling in aging and in age-related neurodegenerative disorders.

## 1. Aging Is the Most Important Factor for Neuronal Damage

Aging is frequently associated with cognitive decline and an increased risk of neuronal damage associated with stroke or neurodegenerative diseases. Specifically, aging is considered the most critical factor for stroke [1] and drastically decreases the rate of survival after stroke [2]. In animal models of stroke, recovery after middle cerebral arterial occlusion decreases dramatically with aging [3], and consistently with the view of enhanced susceptibility to brain damage with age. One of the mechanisms leading to neuron cell death during stroke is excitotoxicity [4]. Excitotoxicity also contributes to cognitive decline in aging and in neurodegenerative diseases, including Alzheimer’s disease (AD) [5,6]. Neuronal damage induced by excitotoxicity in the elderly has been related to age-related changes in the expression of glutamate receptors, particularly Ca^2+^ permeable receptors activated by N-methyl-D-aspartate (NMDA). Consistently, NMDA receptor (NMDARs) antagonists as, for instance, Memantine, partially inhibit NMDARs and the massive Ca^2+^ influx linked to NMDA receptor activation, thus protecting neurons against damage. In fact, Memantine is one of the few drugs approved for neurodegenerative diseases treatment, such as AD. Neuronal damage linked to intracellular Ca^2+^ homeostasis has been also related to neuroinflammation due to pathogen-associated molecular patterns and endogenous ligands known as damage-associated molecular patterns released upon cell damage and necrosis. Consistently, several non-steroidal anti-inflammatory drugs (NSAIDs) may help preventing the cognitive decline associated with aging [7]. Remarkably, the mechanisms involved do not seem to be related to the anti-inflammatory activity of the above over the counter compounds. 

## 2. Intracellular Ca^2+^ Dyshomeostasis Is Involved in Neuronal Damage

Intracellular free Ca^2+^ concentration is involved in the control of many physiological functions in all cell types, and neurons are not an exception in this regard. Rises in cytosolic Ca^2+^ levels are involved primarily in neurotransmitter release, which takes place after activation of voltage-dependent Ca^2+^ channels following depolarization; and/or after activation of inositol triphosphate receptors (IP_3_Rs) and/or Ca^2+^-sensitive ryanodine receptors, which releases Ca^2+^ from intracellular stores. In addition, Ca^2+^ is also involved in synaptic plasticity that depends on NMDARs activation [8]. Synaptic plasticity also relies on spine stability that is modulated by store-operated Ca^2+^ entry (SOCE), mediated by Orai1 channels [9]. In the absence of such entry, mushroom spines are not stable, leading to impaired synaptic plasticity. 

In addition to these critical physiological functions, an excess of Ca^2+^ entry—or Ca^2+^ overload—leads to neuron cell death. For instance, excess of glutamate and/or K^+^ in the extracellular medium, two conditions that may concur during stroke, overactivate both NMDARs and voltage-gated Ca^2+^ channels leading to an excess of Ca^2+^ entry. This excess is managed in healthy neurons by activating energy consuming Ca^2+^ pumps that clear Ca^2+^ loads efficiently but slowly, intracellular Ca^2+^ buffers, and mitochondria. 

Mitochondria act as sinks of Ca^2+^ loads that quickly and efficiently remove Ca^2+^ from intracellular Ca^2+^ hot spots without consuming energy. This is possible because of the mitochondrial Ca^2+^ uniporter (MCU), a Ca^2+^-activated Ca^2+^ channel that transports Ca^2+^ efficiently down the huge mitochondrial membrane potential (∆Ψm) [10,11]. ∆Ψm is negative inside the mitochondrial matrix and is the electromotive force for mitochondrial Ca^2+^ uptake. Formerly, it was believed that mitochondria simply act as Ca^2+^ stores. However, more recently, it has been established that the Ca^2+^ taken up by mitochondria exits back to the cytosol in exchange for Na^+^ through the mitochondrial Na^+^/Ca^2+^ exchanger (NCLX), or in exchange for H^+^ through the mitochondrial H^+^/Ca^2+^ exchanger. However, if Ca^2+^ signals are exceedingly larger and/or more sustained than certain values, the mitochondrial extrusion mechanisms are not able to extrude Ca^2+^ at a consistent rate, leading to mitochondrial Ca^2+^ overload. This process induces the opening of the mitochondrial permeability transition pore (mPTP), a channel complex located at the interface between the inner and the outer mitochondrial membranes [12], resulting in the release of cytochrome c and other pro-apoptotic proteins to the cytosol. This is a point of no return for apoptosome activation irreversibly leading to apoptotic cell death. This process is also favored by an excess of reactive oxygen species (ROS), loss of mitochondrial membrane potential, and low ATP levels [13]. Accordingly, all the mechanisms that promote excessive Ca^2+^ entry, Ca^2+^ release, and/or lack of intracellular Ca^2+^ buffering may contribute to Ca^2+^-dependent cell death or increased susceptibility to cell death. This scenario is often favored in different pathophysiological situations, such as excitotoxicity, neuroinflammation, aging, and/or neurodegenerative disorders, where some neurotoxins involved in Ca^2+^ overload may accumulate, including glutamate, aspartate, K^+^, glycine, and amyloid β peptide species.

Additionally, an increased firing of neurons has also been linked to aging in dopaminergic neurons, caused by a remodeling of voltage-gated Ca^2+^ channels [14]. Moreover, aging also depends on the depletion of intracellular Ca^2+^ buffers in specific brain regions. For example, loss of calbindin with aging has been suggested to contribute to the increased susceptibility to Ca^2+^ overload in hippocampal neurons in AD [15].

## 3. Long-Term Culture of Rat Hippocampal Neurons as an In Vitro Model of Neuronal Aging

It has been previously reported that immature cultured neurons are resistant to glutamate- and NMDA-induced neurotoxicity [16,17,18,19]. Interestingly, this vulnerability to glutamate excitotoxicity increases with the days in culture. Therefore, the longer the time of the neuronal culture, the larger the damage induced. These effects seem to be related to changes in the expression level of NMDARs and their subunit composition [20]. Along these lines, it has been shown that rat hippocampal neurons cultured for several weeks exhibit some of the typical hallmarks commonly associated with neuronal aging [21]. As a matter of fact, after approximately three weeks in vitro, hippocampal neurons exhibit many of the typical hallmarks accompanying neuronal aging in vivo, including accumulation of ROS, lipofuscin granules, heterochromatic foci, and activation of the Jun N-terminal protein kinase (pJNK) and p53/p21 pathways. Brain cholesterol levels decrease with age in the human brain and in mouse hippocampus. This reduction has also been observed in embryonic hippocampal neurons aging in culture [21]. This cellular model of in vitro aging has been used to investigate the possible cause of age-accompanying loss of cholesterol. Therefore, a long-term culture of rat hippocampal neurons has been proposed as an in vitro model of neuronal aging, which allows us to study of the remodeling of Ca^2+^ signaling longitudinally with aging. 

## 4. Effects of Neurotoxins in Short-Term and Long-Term Cultures of Rat Hippocampal Neurons

The effects of several neurotoxins, such as the glutamate receptor agonist NMDA or the amyloid β peptide, have been tested in short-term and long-term cultures of rat hippocampal neurons resembling young and aged neurons respectively, and their toxic effects on cell death and Ca^2+^ signaling among others have been compared between the two groups.

NMDA did not induce cell death in hippocampal neurons cultured for only two days in vitro (2 DIV), considered young neurons. After 8 DIV, NMDA slightly but significantly increased the rate of neuronal apoptosis from about 5% to 10%. However, this effect dramatically increased at 13 to 21 DIV, considered aged neurons, in which NMDA exposure increased apoptosis from about 12% to 60–80%. Thus, long-term cultured neurons reflecting aged neurons were highly sensitive to excitotoxicity [22]. 

Interestingly, the rises in intracellular Ca^2+^ concentrations induced by NMDA were very small or nearly negligible at 2 DIV, significantly increased at 8 DIV, and became exceedingly large at 13 to 21 DIV, establishing a correlation between the NMDA-induced Ca^2+^ increase and the apoptosis rate. The Ca^2+^ rises induced by plasma membrane depolarization with high K^+^ concentration medium also increased with culture time but the difference was smaller [22]. These results suggest that in vitro aging is associated with an enhanced expression of NMDARs. Data obtained using semi-quantitative immunofluorescence indicated that in vitro aging is associated with an increased expression of NR1 and NR2A subunits of the NMDAR, and decreased expression of NR2B subunit [22]. Interestingly, this pattern of changes resembles the pattern reported in in vivo aging [21]. 

Accordingly, enhanced Ca^2+^ entry mediated by an increased expression of NMDARs could be involved in the enhanced susceptibility to neuronal damage in aged neurons. This possibility is supported by additional evidence showing that NMDA does not promote mitochondrial Ca^2+^ uptake in short-term cultured rat hippocampal neurons [22]. However, at 8 DIV, and particularly at >13 DIV, NMDA promotes large increases in mitochondrial Ca^2+^ concentration ([Ca^2+^]_mit_). Consistently, inhibiting the driving force for mitochondrial Ca^2+^ uptake (loss of ∆Ψm) with low concentrations of mitochondrial uncouplers, such as FCCP, or several NSAIDs acting as mild mitochondrial uncouplers, prevented NMDA-induced mitochondrial Ca^2+^ uptake and neuron cell death [22]. Taking this into account, we can conclude that aging is associated with enhanced susceptibility to cell death due to Ca^2+^ channel remodeling, at least in vitro. This remodeling depends on changes in the expression of NMDAR subunits, enhanced Ca^2+^ entry and mitochondrial Ca^2+^ overload. In addition, inhibiting this mitochondrial Ca^2+^ overload protects from neuronal cell death. Interestingly, this mechanism may explain the anti-inflammatory-independent mechanism of neuroprotection afforded by low concentrations of NSAIDs [23]. 

AD is the neurodegenerative disorder most commonly associated with age, which implies neuronal cell death and progressive cognitive impairment. Even though it is one of the most prevalent dementias, the direct cause of AD is still unknown. It has been proposed that soluble low molecular weight aggregates (oligomers) of the amyloid β peptide (Aβo) are the main neurotoxin in AD. Aβo promote Ca^2+^ entry into the cytosol and mitochondrial Ca^2+^ uptake in rat cerebellar granule cells and hippocampal neurons leading to mitochondrial Ca^2+^ overload and apoptosis [24]. These effects have been partially attributed to activation of NMDA receptors by Aβo [25]. However, it has been claimed that some of these results are related to glutamate contamination of the media in which the synthetic Aβo were prepared [26,27]. Nevertheless, further results using Aβo prepared in media devoid of glutamate receptor agonists confirmed that Aβo promote Ca^2+^ entry and mitochondrial Ca^2+^ overload in primary cultures of both rat cerebellar neurons and hippocampal neurons [24,27]. 

Aβ itself has been hypothesized to form Ca^2+^-permeable pores or non-specific ion channels in the plasma membrane [28,29]. On the other hand, presenilins (PSs), the catalytic core of γ-secretase which participates in the amyloid precursor protein (APP) processing, have been proposed to be an ER Ca^2+^-leak channel [30], independently of their catalytic activity in the γ-secretase. It has been shown that mutations in PSs can disturb Ca^2+^ release through the leak channels, leading to an increase of Ca^2+^ in the ER and increasing the vulnerability to neurodegeneration [31]. Therefore, Aβ pores and/or Aβ-activated NMDA receptors, together with mutant PS channels may all lead to severe general Ca^2+^ overload in genetic cases of AD. 

Interestingly, Aβo had no effect on apoptosis in short-term cultures of rat hippocampal neurons, but started to promote cell death at 8 DIV and, after longer culture periods (>13 DIV), oligomers caused apoptosis in more than 40% of the neurons as assessed by fluorescence imaging [32]. Consistently, Aβo induced no rise in cytosolic Ca^2+^ concentration in young neurons, but started to increase cytosolic Ca^2+^ at 8 DIV, and the effects increased further in aged neurons at >13 DIV. These effects were observed only in neurons but not in glial cells extracted from rat hippocampi, as demonstrated by simultaneous Ca^2+^ imaging and immunofluorescence in the same individual cells. Moreover, Aβo promoted no rise in mitochondrial Ca^2+^ concentration in short-term cultures of rat hippocampal neurons. However, Aβo induced a small rise in mitochondrial Ca^2+^ at 8 DIV, and the effects increased rather dramatically in aged neurons at >13 DIV [32]. In addition, Aβo promoted cytochrome c release only in in vitro aged neurons but not in the short-term neurons. 

Interestingly, inhibition of mitochondrial Ca^2+^ uptake using low concentrations of mitochondrial uncouplers or several NSAIDs prevented apoptosis induced by Aβo without having any effect on cytosolic Ca^2+^ [32]. The effects of NSAIDs are mimicked by R enantiomers, like R-flurbiprofen, that lack anti-inflammatory activity, indicating that neuroprotection relates to NSAID’s ability to inhibit mitochondrial Ca^2+^ overload rather than to their anti-inflammatory activity. However, it must be emphasized that neuroprotection is only achieved at low NSAID concentrations that depolarize mitochondria only partially. At large concentrations, NSAIDs collapse mitochondrial potential and induce apoptosis even in the absence of Aβo [32]. These data indicate that the vulnerability of rat hippocampal neurons to Aβo depends strongly on the time of culture related to the phenotypic age of the neurons. They also point to mitochondrial Ca^2+^ as a key player in the Aβo-induced neurotoxicity. In addition, they provide strong evidence that NSAIDs may protect against Aβo toxicity and perhaps AD by preventing the associated mitochondrial Ca^2+^ overload. 

Unfortunately, latest trials with NSAIDs aimed at preventing or treating AD have given rather pessimistic results. In the last trial carried out in cognitively intact individuals at risk (INTREPAD), the sustained treatment with naproxen sodium increased the frequency of adverse health effects but did not reduce apparent progression of pre-symptomatic AD [33]. Further research is required to ascertain discrepancies with previous studies. 

AD has also been related to neuroinflammation. The AD brain shows increased levels of pro-inflammatory factors, such as pro-inflammatory cytokines, complement components, and proteases, which are recognized by different receptors in glial cells and neurons. Neuroinflammation comprises a set of cellular and molecular responses, and recent evidence suggests that Ca^2+^ signaling is also involved in this regard. Specifically, toll-like receptors (TLRs) are transmembrane pattern-recognition receptors of the innate immune system that recognize several pathogen-derived and tissue damage-related ligands. It has been proposed that TLR signaling may contribute to age-related neurodegenerative diseases, including AD, suggesting a possible interplay between inflammation and Aβo in AD [34]. Consistent with this view, it has been recently shown that lipopolysacharide (LPS), an agonist of TLR4, increases cytosolic Ca^2+^ and promotes apoptosis in long-term cultures of rat hippocampal neurons but not in short-term cultured neurons [35]. Both, the increase in cytosolic Ca^2+^ and neuronal apoptosis induced by LPS were prevented by the TLR4 antagonist CAY10614. Interestingly, hippocampal neurons express TLR4 receptors and the expression level increases with time in culture consistently with the rise in TLR4 in aging brains [36]. Furthermore, chronic exposure of aged hippocampal cultures to Aβo further increased TLR4 expression and enhanced cytosolic Ca^2+^ rises and apoptosis induced by LPS. These results suggest that in vitro aging increases the susceptibility of rat hippocampal neurons to LPS-induced damage, and these effects seem to be related to changes in the TLR4 expression [35]. 

Altogether, these data suggest that rat hippocampal neurons in long-term culture may be a good model to investigate age-related changes in susceptibility to neurotoxins related to excitotoxicity, AD, and neuroinflammation. 

## 5. Remodeling of Intracellular Ca^2+^ and Store-Operated Ca^2+^ Entry (SOCE) in In Vitro Aged Neurons 

Data suggest that long-term cultured neurons resume many of the characteristics of aged neurons, at least regarding susceptibility to neuron damage and changes in expression of receptors targeted by neurotoxins related to age-dependent neurodegenerative processes and Ca^2+^ signaling. Therefore, changes in intracellular Ca^2+^ homeostasis (Ca^2+^ remodeling) could be involved in the aging process. These changes have recently been explored in the model of a long-term culture of rat hippocampal neurons. 

One of the characteristics of cultured hippocampal neurons is that they connect each other forming neural networks. This is evidenced by their ability to display spontaneous and synchronous intracellular Ca^2+^ oscillations after a few days in vitro, when neurons express mature NMDARs [36,37]. These oscillations can be abolished by tetrodotoxin, indicating they are dependent on synaptic network communication among neurons. However, after several weeks in vitro, the neuronal cultures lose this ability and show increased resting levels of intracellular Ca^2+^ concentration relative to the short-term cultures instead [38]. Chronic exposure of rat hippocampal neurons to Aβo for 24 h has no effect on synchronic cytosolic Ca^2+^ oscillations. Intriguingly, in aged neurons, Aβo treatment resumes synchronic oscillations [38]. Ca^2+^ hyperactivity has also been shown in in vivo mouse models of cerebral amyloidosis [39]. The functional consequences of this effect are not clear, but they provide evidence that aged neurons are sensitive to Aβo whereas young neurons are not. 

SOCE is an important Ca^2+^ entry pathway activated after Ca^2+^ release from the ER induced by physiological agonists such as acetylcholine or glutamate activating metabotropic receptors. SOCE is involved in many different physiological functions in most cell types [40]. In neurons, the physiological role of SOCE is not well known. Recent data provided by several laboratories indicate that SOCE could be critical for the stabilization of mushroom spines involved in memory storage. Importantly, mushroom spines’ stability is downregulated in age-related neurodegenerative disorders, such as AD and Parkinson’s disease [36]. At the molecular level, SOCE is activated by the interaction of an ER Ca^2+^ sensor, the stromal interacting molecular 1 (STIM1), with a plasma membrane channel, Orai1. Other isoforms of these proteins, including STIM2, Orai2, and Orai3, can also be involved. Rat hippocampal neurons display SOCE and express both Orai1 and STIM1, the classic molecular players involved in SOCE. Interestingly, the amplitude of SOCE in rat hippocampal neurons decreases significantly with culture time [37]. Specifically, the rise in cytosolic Ca^2+^ induced by extracellular Ca^2+^ addition to cultured neurons pre-treated with thapsigargin (commonly used to deplete intracellular Ca^2+^ stores) is easily recorded in short-term cultures of rat hippocampal neurons, and decreases significantly in long-term cultures [37]. Loss of SOCE is associated with a decreased expression of both Orai1 and STIM1 in aged neurons. Interestingly, exposure to Aβo has no effect on SOCE in the short-cultured ones. However, Aβo treatment decreases SOCE to even lower levels in the long-term cultures [38]. Accordingly, it is conceivable that a loss of SOCE in aged neurons may contribute to a cognitive decline in the elderly, particularly in excess of Aβo, a scenario observed in AD. Notice that Aβo may be present in the brain of patients for decades; however, damage is only evident as they age, suggesting the interesting possibility that it is the combination of Aβo excess and the age-associated remodeling of intracellular Ca^2+^ homeostasis which is harmful for neurons. If this is confirmed, then the reversal of Ca^2+^ remodeling could be an efficient novel approach for AD prevention. 

## 6. Remodeling of Ca^2+^ Store Content and Ca^2+^ Release in In Vitro Aged Neurons 

**Figure 1:**
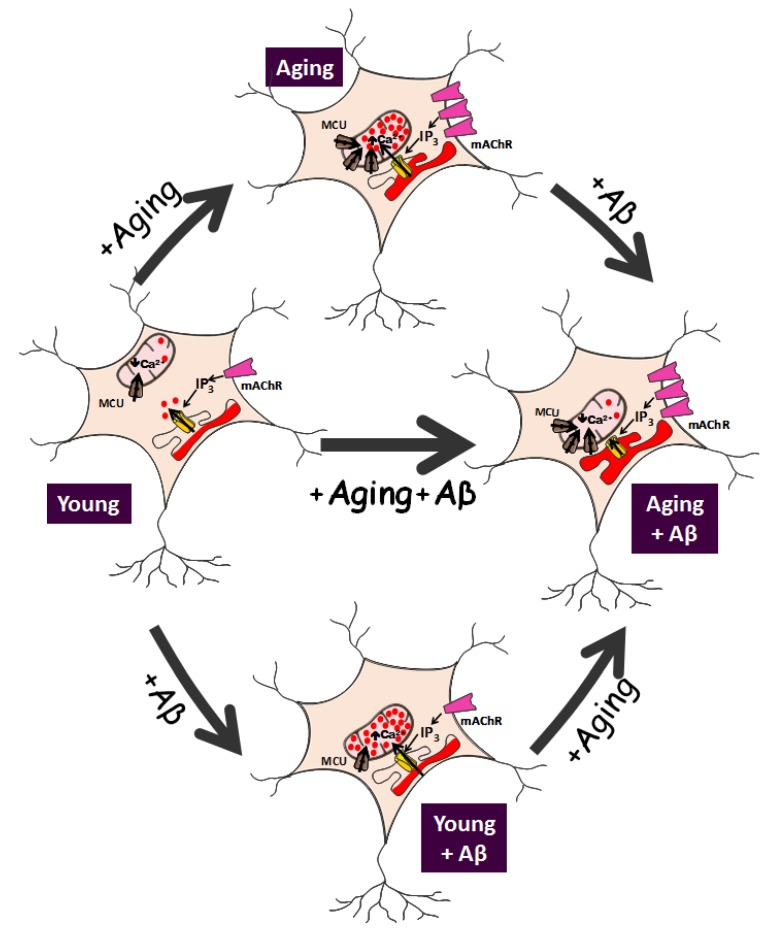
Intracellular Ca^2+^ remodeling in in vitro aged rat hippocampal neurons and effects of amyloid oligomers. Small arrows depict Ca^2+^ fluxes from the endoplasmic reticulum (ER) to mitochondria, increases or decreases in intracellular Ca^2+^ concentration and IP_3_ signaling. Large arrows outside neurons represent phenotypic remodeling from young neurons induced by aging, amyloid oligomers (Aβ) or both. In vitro aging enhances Ca^2+^ store content, Ca^2+^ release, and Ca^2+^ transfer from ER to mitochondria associated in cultured rat hippocampal neurons. The remodeling is associated with changes in expression of metabotropic acetylcholine receptors (mAChR), inositol triphosphate (IP_3_) receptors, and mitochondrial Ca^2+^ uniporter (MCU), thus improving energy production at the expense of an increased risk of mitochondrial Ca^2+^ overload. Treatment of young neurons with amyloid β oligomers (Aβo) enhances ER–mitochondria colocalization and Ca^2+^ transfer from ER to mitochondria without deleterious effects. However, Aβ treatment in aged neurons showing Ca^2+^ remodeling disrupts mitochondrial Ca^2+^ homeostasis leading to apoptosis.

As stated above, mitochondrial Ca^2+^ uptake is critically involved in neuronal apoptosis. Due to the low Ca^2+^-affinity of the MCU, mitochondria take up Ca^2+^ from microdomains, which exhibit high Ca^2+^ concentrations and are typically formed at the inner mouth of plasma membrane Ca^2+^ channels [41]. In addition, mitochondria also sense high Ca^2+^ domains formed nearby IP_3_ receptor channels in the ER, particularly those located at the interface between the ER and mitochondria, at the mitochondria-associated membranes, the so-called MAMs [42]. Accordingly, the Ca^2+^ content of intracellular Ca^2+^ stores, the extent of Ca^2+^ release though the IP_3_ receptors, and the resulting Ca^2+^ uptake by mitochondria are key parameters of intracellular Ca^2+^ homeostasis in neurons. Recent data indicate that in vitro aging is associated with changes in several parameters consistently with an age-related remodeling of intracellular Ca^2+^ homeostasis [37,38] [Figure 1]. Specifically, Ca^2+^ store content increases with time in culture. This has been shown by the size of the rise in cytosolic Ca^2+^ induced by low concentrations of the Ca^2+^ ionophore ionomycin that release Ca^2+^ from intracellular stores [37]. Consistently, caffeine (an agonist of ryanodine receptors) and acetylcholine, that promotes IP_3_ synthesis and the ensuing activation of IP_3_ receptors, induce a Ca^2+^ release from the ER that is significantly enhanced in aged cultures compared to young cultures [37]. Therefore, ER Ca^2+^ release is enhanced in aging neurons. This may be a logic consequence of enhanced resting intracellular Ca^2+^ levels in aged cells as mentioned above, but this may be also mediated by changes in the expression of IP_3_ receptors. In fact, immunofluorescence imaging using specific antibodies against different IP_3_ receptor subunits suggests that the expression of all three IP_3_ receptor subunits (IP_3_R1, IP_3_R2, and IP_3_R3) is enhanced in aged neurons relative to young neurons [38]. This provides an additional mechanism for enhanced ER Ca^2+^ release in aged neurons. Interestingly, it has also been reported that Aβo treatment enhances further Ca^2+^ store content and Ca^2+^ release induced by acetylcholine or caffeine. Again, these effects are only observed in aged cultures of rat hippocampal neurons but not in young cultures [38]. Therefore, in vitro aging increases Ca^2+^ store content and Ca^2+^ release from intracellular stores, and these effects are further potentiated by Aβo treatment (Figure 1). 

## 7. Remodeling of Ca^2+^ Transfer from ER to Mitochondria Induced by Aβ and Aging 

A functional consequence of enhanced Ca^2+^ store content and Ca^2+^ release in aged neurons could be enhanced Ca^2+^ transfer from ER to mitochondria. The ER–mitochondria coupling is gaining momentum particularly because of the discovery of MAMs, specialized junctions of interaction between the ER and mitochondria [42]. Ca^2+^ transfer from ER to mitochondria has been investigated also longitudinally in cultures of rat hippocampal neurons. 

Specifically, Calvo-Rodriguez et al. [37] showed no or little Ca^2+^ transfer from ER to mitochondria in young cultures of rat hippocampal neurons. When stimulating hippocampal neurons with acetylcholine to release Ca^2+^ from the ER, the rise in cytosolic Ca^2+^ was similar in young cultures in normal conditions or in presence of a mitochondrial uncoupler to prevent mitochondrial Ca^2+^ uptake [37]. However, in aged cultures, the rise in cytosolic Ca^2+^ induced by acetylcholine increased dramatically if mitochondria were not able to take up Ca^2+^ (following pre-treatment with the mitochondrial uncoupler). The above evidence indicates that, in contrast to young neurons, most of the Ca^2+^ released by the ER is taken by the surrounding mitochondria in the aged neurons [37]. This view is further supported by direct measurements of mitochondrial Ca^2+^ uptake using bioluminescence imaging of individual neurons transfected with mitochondria-targeted aequorin [43]. In this sense, Ca^2+^ release after acetylcholine stimulation induced no or very little mitochondrial Ca^2+^ uptake in young cultures. On the contrary, it induced large mitochondrial Ca^2+^ uptake in the aged cultures [37]. Therefore, in vitro neuronal aging is associated with a deep remodeling of the coupling ER–mitochondria that favors Ca^2+^ transfer from the ER to mitochondria [Figure 1]. 

Several mechanisms could contribute to the enhanced transfer of Ca^2+^ from ER to mitochondria in the aged neurons. The first one has been mentioned above: increased ER Ca^2+^ content together with enhanced ER Ca^2+^ release. Changes in expression of acetylcholine, IP_3_, and ryanodine receptors could contribute to these changes. As stated above, immunofluorescence of cultured neurons suggests upregulation of all three IP_3_ receptor subunits in long-term cultures [38]. Second, evidence from confocal microscopy imaging analysis of tagger ER and mitochondria suggests that ER–mitochondria colocalization is enhanced in long-term cultures of rat hippocampal neurons [38], which could also contribute to the enhanced Ca^2+^ transfer between these two organelles. 

Mitochondria may also contribute to the aging-induced enhanced Ca^2+^ transfer ER–mitochondria. As stated above, mitochondrial Ca^2+^ uptake depends on MCU and ∆Ψm. Recent evidence indicates that MCU expression increases with time in culture, suggesting that MCU is upregulated in aged hippocampal neurons [37]. These data may explain the increased mitochondrial Ca^2+^ uptake in aged cultures described above. However, since Ca^2+^ release from the ER is also enhanced in aged neurons, the specific contribution of each mechanism cannot be distinguished. This was analyzed in detail using permeabilized neurons subjected to a clamped cytosolic Ca^2+^ concentration. In this experimental setting, mitochondrial Ca^2+^ uptake was paradoxically lower in aged neurons compared to young neurons. This cannot be attributed to a decreased MCU expression. Instead, it could be explained by the partial loss of ∆Ψm reported in aged neurons [37]. Therefore, these data suggest that long-term cultured hippocampal neurons display an enhanced ER–mitochondria Ca^2+^ transfer mediated by several mechanisms mostly related to enhanced ER Ca^2+^ release and increased ER–mitochondria colocalization in aged neurons in culture [Figure 1]. 

What are the functional consequences of enhanced Ca^2+^ transfer from ER to mitochondria in aged neurons? It may favor the activation of Ca^2+^-dependent dehydrogenases involved in the Krebs cycle, thus supporting ATP synthesis in metabolically compromised aged neurons. However, this improvement of energy production increases the risk of mitochondrial Ca^2+^ overload and apoptosis. In consequence, the remodeling of intracellular Ca^2+^ homeostasis observed in aged neurons in vitro may contribute to explain some of the characteristics of brain aging, including cognitive decline and enhanced susceptibility to neuron damage. 

Ca^2+^ transfer from ER to mitochondria has also been investigated in the context of AD, in cultures of rat hippocampal neurons treated with Aβo. Evidence indicates that a 24 h treatment with Aβo increases the ER–mitochondria Ca^2+^ transfer. This effect is observed in short-term cultures of rat hippocampal neurons [Figure 1]. Remarkably, this is the only effect induced by Aβo in young neurons. It is important to bear in mind that, as stated above, Aβo have no effect on resting cytosolic Ca^2+^, SOCE, Ca^2+^ store content or ER Ca^2+^ release in young cultures. Therefore, Aβo-enhanced Ca^2+^ transfer from ER to mitochondria cannot be attributed to possible effects on Ca^2+^ store content, Ca^2+^ release, or changes in expression of MCU since Aβo treatment in young neurons does not influence any of the above mentioned parameters. However, Aβo treatment also increases ER–mitochondria colocalization in young cultures. Therefore, these data imply that Aβo increase ER–mitochondria Ca^2+^ transfer most likely by promoting the colocalization of these two organelles. In fact, it has been proposed that modulation of the ER–mitochondria interface could be “the” physiological role of Aβo [38]. This function may be beneficial in young neurons by improving the coupling ATP synthesis rate to energy demands after cell stimulation. In fact, Aβo decrease partially mitochondrial membrane potential in young neurons consistently with enhanced respiration and metabolic activity [38]. Nevertheless, Aβo effects are not deleterious to young neurons as evidenced by the lack of apoptosis induced by Aβo. The reason for this effect might be that, despite increased Aβo-induced ER–mitochondria colocalization, mitochondrial Ca^2+^ overload is limited in this scenario by the low Ca^2+^ store content, the small Ca^2+^ release, and the low expression levels of IP_3_ receptors and MCU. 

It is not entirely clear how a peptide such as Aβ could mechanistically affect the intracellular localization of ER–mitochondria and increase their interaction. Area-Gomez et al. first found increased ER–mitochondrial contacts in AD patient fibroblasts and PS-mutant cells [44]. Additionally, they recently proposed that C99 (a terminal fragment generated after the cleavage by β-secretase of the amyloid precursor protein (APP) that is then cleaved by γ-secretase to produce Aβ) is found in the MAMs, and its localization is increased in AD, causing mitochondrial dysfunction. Importantly, uncleaved C99 in the MAMs induces both physical and functional enhancement of ER–mitochondrial connections [45]. Along these lines, by using pure mitochondrial fractions, Ankarcrona’s group recently proposed that Aβ peptides are generated in the MAMs, which may disturb ER, mitochondria, and ER–mitochondria contact site function if the peptide is produced in excess [46].

Aβo effects on apoptosis may be quite different in the aging scenario. Aged neurons do display enhanced Ca^2+^ store content, large Ca^2+^ release after stimulation, and increased expression of IP_3_ receptors and MCU. Surprisingly, in aged neurons treated with Aβo, Ca^2+^ transfer from ERto mitochondria is rather abolished [38]. This effect is observed despite that ER–mitochondria colocalization is enhanced in aged cells, and increased even further after Aβo treatment [Figure 1]. However, as stated above, mitochondrial membrane potential is quite low in aged cells, and further decreased by Aβo treatment. This scenario of very low mitochondrial membrane potential, ER Ca^2+^ overload, and enhanced ER–mitochondrial coupling may favor failure of mitochondrial Ca^2+^ homeostasis and mPTP opening, leading to apoptosis [38]. Further research is required to confirm this evidence in vivo. However, if this is true, then the combination of age-related, intracellular Ca^2+^ remodeling with the physiological effect of Aβo increasing ER–mitochondria coupling may be critically involved in the catastrophic consequences of neuronal damage in AD. Some of the evidences reviewed here should be confirmed in in vivo aging. However, the preparation of long-term cultures of rat hippocampal neurons as a model of aging [37,38] is worthy of consideration when it comes to investigating functional and molecular changes related to aging. Finally, we would like to emphasize that in this context, the pharmacological modulation of Ca^2+^ fluxes, particularly at the ER–mitochondria interface, should be considered further for fighting cognitive decline and neurodegenerative disorders in the elderly. 

## 8. Concluding Remarks

Aging is a highly complex, physiological process in which cell, organ, and whole organism dysfunctions are prone to accumulate. In spite of its complexity, some of the characteristics of healthy aging are observed in cells cultured for long periods of time. Evidence indicates that this may happen in long-term isolated rat hippocampal neurons in primary culture. First, cells acquire characteristics of differentiated neurons, express high levels of neurotransmitter receptors, and connect to other neurons to form neural networks with spontaneous synchronic activity. After more than two weeks in culture, neurons start displaying many characteristics of senescent or aged cells, and the expression of aging markers increase. These phenotypic changes are associated with changes in intracellular Ca^2+^ homeostasis that prevent spine stability and increase the susceptibility to cell death induced by different insults. These changes include loss of store-operated Ca^2+^ channels involved in spine stability and enhanced Ca^2+^ functional coupling between the ER and mitochondria. At the functional level, Ca^2+^ remodeling favors energy production at the expense of increased susceptibility to apoptosis induced by mitochondrial Ca^2+^ overload. Treatment of cultured cells with Aβo enhances this coupling in young neurons. However, in aged neurons, enhanced coupling induced by oligomers disrupts mitochondrial Ca^2+^ homeostasis and mitochondrial Ca^2+^ buffer capacity. Further research is required to ascertain whether these changes may contribute to a cognitive decline and enhanced susceptibility to cell death in aged neurons in vivo.
